# Words matter: the prevalence of chiropractic-specific terminology on Australian chiropractors’ websites

**DOI:** 10.1186/s12998-020-00306-9

**Published:** 2020-04-07

**Authors:** Kenneth J. Young

**Affiliations:** School of Sport and Health Sciences, University of Central Lancashire, Preston, PR1 2HE England

**Keywords:** Chiropractic, Language, Cultural authority, Evidence-based practice

## Abstract

**Background:**

Chiropractors use words and phrases in unique ways to express traditional, chiropractic-specific theories. This lexicon represents concepts that reinforce the separation of chiropractic from other health care professions. It may impact referrals both to and from chiropractors, lead to public confusion about health care issues, and reduce cross-disciplinary research. Therefore, it is important to understand how prevalent chiropractic-specific terms are in publicly available media.

**Methods:**

Five chiropractic terms were selected: subluxation, adjustment, vital (−ism/−istic), wellness, and Innate (Intelligence). States and territories in Australia were proportionately sampled according to population of chiropractors using a Google search for chiropractors’ private practice websites. The top results were recorded. Websites were word-searched on every publicly available page for the five terms. Context was checked to count only terms that were used to support a chiropractic-specific concepts. The number of occurrences of each term was recorded, tallied nationally and by state/territory. Descriptive statistics were applied to determine prevalence.

**Results:**

Three hundred sixty-nine websites were sampled, based on an estimate of 5500 chiropractors practising in Australia. Nationally, 85% of chiropractors used one or more terms. The term adjust (−ing/−ment) occurred most frequently, being found on 283 websites (77%) with a total of 2249 occurrences. Wellness was found on 199 websites (54%) with 872 occurrences; subluxation was found on 104 websites (28%), 489 occurrences; vital (−ism/−istic) on 71 websites (19%) with 158 occurrences; and Innate was least used, being found on 39 websites (11%) with 137 occurrences.

**Conclusion:**

A majority of the Australian chiropractors sampled used one or more chiropractic-specific terms on their websites. Future research should explore the effects of chiropractic language on the public, policy-makers, and other health care professionals.

## Background

Use of a common language is essential to mutual understanding. This sentiment has been noted in a variety of fields, from dance education to corporate governance to disability, and more [1–5]. The chiropractic profession uses words and phrases in a unique way to express traditional chiropractic-specific theories and concepts [6, 7]. This lexicon reinforces the separation of chiropractic from other health care professions [8, 9]. Promoting chiropractic as alternative rather than complementary or mainstream may be detrimental to patients by deterring or delaying referrals both to and from other health care professionals [10–14]. Barker, Reid, and Lowe [15] listed three different reasons common language was important when communicating information about back pain: 1) patients seeking information from health care professionals may encounter difficulty interpreting health care literature, 2) misunderstandings may arise among health professionals, and 3) a lack of standard definitions can make comparison of research studies challenging. Lawrence noted that semantic incompatibilities have hampered intraprofessional development of chiropractic as well [16]. Authors have called for the abandonment of traditional, uniquely chiropractic language [17]. Therefore, it may be useful to learn how prevalent chiropractic-specific terms are in publicly available advertising media, such as websites. Five terms stood out in the chief investigator/author’s opinion as likely to express uniquely chiropractic concepts. They were: subluxation, adjust (−ing/−ment), vital (−ism/−istic), wellness, and Innate (Intelligence). Subluxation and adjustment have been shown to be prevalent in chiropractic literature [18].

### Subluxation

Subluxation is a term used in medicine to denote a partial dislocation, a significant injury to a joint, involving damage to soft tissues and possibly articular cartilage and bone [19]. In chiropractic the definition is different, indicating a tiny displacement of one bone in relation to another, possibly affecting the function of a nearby nerve or nerves [20]. The chiropractic version has no profession-wide agreement, has poor inter- and intra-observer reliability, and is sometimes claimed, with little evidence, to cause a wide range of diseases [21].

Several papers in recent years have examined the prevalence and use of the word subluxation by chiropractors. In 2019, Marcon, Murdoch, and Caulfield studied Alberta, Canada [22]. They found that 121 clinic websites (33%) presented a theory for chiropractic subluxation and claimed wide-ranging health benefits from its removal. In 2018, Funk, et al. gathered data from 46 chiropractic courses around the world and tallied the number of instances of the word subluxation in course/unit/module titles and descriptions but did not analyse the context [23]. So even websites that used the term in a non-positive sense, for example to dissociate themselves from that line of thinking, were counted in the study even though the meaning of the term was not being used to support or advocate traditional chiropractic concepts. Funk’s study counted the word subluxation no matter in what context it was used. For instance, ‘We practice subluxation-based chiropractic’ and ‘We believe subluxation is a historical concept only’ would both have been counted. This paper followed on from a 2011 study by Mirtz and Perle which examined course/unit/module descriptors in North American chiropractic teaching institutions [24]. The latter study found an increase in the mean incidence of the term, rising from 5.53 in the first study to 6.50 in the follow-up at the North American Institutions. Funk found lower incidence of the ‘subluxation’ outside North America (mean of 0.83) [23].

### Adjust (−ing/−ment)

The use of a chiropractic-specific meaning of adjust originates with DD Palmer: “The science of chiropractic has led to the creation of the art of vertebral adjusting.” [25] The Cambridge English dictionary defines adjustment as, “a slight change made to something to make it fit, work better, or be more suitable” [26]. Mirriam-Webster online offers as one of its definitions a specific chiropractic variant: “the manual or mechanical manipulation of a joint (especially the spine) in which a controlled force is applied to the joint” [27]. Mirriam-Webster also provides a specific medical definition of manipulate: “to examine or treat by skilful use of the hands, as in palpation, reduction of dislocations, or changing the position of a fetus [sic]” [28]. Therefore, adjustment as used by chiropractors in relation to treatment is a chiropractic-specific term, while the more generic or mainstream term for manually-induced motion of bones and joints used by other health professions is manipulation.

### Vital(−ism/−istic)

Vitalism is not a chiropractic concept per se, but some chiropractors employ a vitalistic paradigm to describe the benefits of chiropractic [29–32]. Sociologist Holly Folk noted that vitalism was found in many alternative nineteenth century health care occupations, but that it has survived in chiropractic [33]. Vitalism is not a concept employed by the medical profession, and it is also not found in chiropractic accreditation education standards [34]. Although there are multiple definitions of vitalism, chiropractors do not adhere to one. Some chiropractors specifically refer to Innate Intelligence, a vitalistic concept, but even this version of vitalism has had multiple definitions. Keating noted that it was a synonym for homeostasis, a label for our ignorance, an “explanation” for as yet poorly understood phenomena, and a metaphysical premise [35]. However many chiropractors only vaguely refer to concepts of a life- or health-giving force [31, 35, 36]. As an entity that is not able to be objectively and directly measurable, it is untestable and therefore not amenable to scientific methods of investigation [37–39]. Use of this term and the concept it represents may result in chiropractic being viewed as an alternative rather than complementary form of health care, thus separating it from the mainstream.

### Wellness

Like vital(−ism/−istic), wellness is not a chiropractic-specific term. While some chiropractors use it in the mainstream way to address public health functions like promoting healthy behaviours (e.g. stop smoking, reduce alcohol consumption), others use it to imply chiropractic-specific benefits theorised to derive from subluxation removal [40–45]. Studies have found that chiropractic wellness-oriented practices tend to overtreat patients and are more likely to have aggressive marketing tactics [46, 47]. In addition, one study has found that chiropractors that practice with a wellness model receive fewer referrals from medical doctors [48].

### Innate (intelligence)

This term was coined by DD Palmer, although it has had several definitions [35, 49]. It is essentially the chiropractic representation of vitalism, and like vitalism, it is often vaguely referred to by chiropractors as an energy- or health-restoring force [50–54]. Its chiropractic uniqueness and metaphysical connotations contribute to chiropractic as separate and somehow different from mainstream health care; some authors also believe it is divisive within chiropractic [38].

The purpose of this paper is to document the prevalence of these five terms on chiropractors’ websites in Australia when they were used to convey or support chiropractic-specific concepts.

## Methods

Sample size was calculated using the Centers for Disease Control tool, Epi Info [55]. The calculations assumed 5500 chiropractors practising in Australia, as well as an expected frequency of 50%, that is, we expected 50% of web sites to contain at least one chiropractic term. A minimum sample of 359 was calculated to provide a 95% confidence interval with a 5% margin of error. Each state/territory was proportionately sampled according to population of chiropractors. The proportion was obtained from the Chiropractic Board of Australia (2108) [56]. Only private practice websites were included, not organisations or educational institutions. We employed the use of the Google search engine to evaluate practice-based websites using the term “chiro” + [name of state or territory] for each state and territory. For instance, we searched for chiro+New South Wales. This resulted in a list of chiropractors’ web sites. We then copied and pasted the URL of the main web page into a Microsoft Excel spreadsheet, starting from the top of each set of results until the required number for that state or territory was collected. For instance, only 1.3% of chiropractors practice in the Australian Capital Territory, which translated to 5 web sites, so the URLs of top 5 websites returned by the Google search were recorded. Then each web site was visited and word-searched on every available page for each of the five terms.

Context was checked to ensure a positive chiropractic meaning for each use of a term. That is, in order to be counted, the term needed to denote a chiropractic-specific context in a positive way. A hypothetical example of a positive meaning is: “We alleviate vertebral subluxations to enhance your health”. An example of a non-positive meaning is: “We view the subluxation concept as historical rather than clinically meaningful”. Positive uses were recorded; non-positive uses were not, because they did not advocate chiropractic uniqueness or separateness. A statement indicating that chiropractic adjustments could directly enhance wellness would have been counted for both the terms adjustment and wellness, as the context denotes a positive, chiropractic-specific meaning. However, the generic use of a term would not, such as: “We believe that your wellness is enhanced when you experience less pain.”

Research assistants were trained in two meetings during which several random chiropractic clinic websites were accessed and the terms found therein discussed among the group, so all reached agreement on positive uses of terms to be recorded, and that negative uses of terms would not be recorded. If a website had both positively-used terms and non-positively-used terms, only the positively-used ones were counted. The research assistants divided the states and territories among themselves and recorded the number of occurrences of each term on the spreadsheet. The chief investigator randomly sampled about 5% of the results submitted by the research assistants to ensure accuracy. No errors or omissions were found. The chief investigator then used Excel mathematical functions to add up the occurrences as well as to determine percentages when compared to the number of practising chiropractors. This gave the prevalence of occurrences by state/territory as well as nationally both by the total number of occurrences of terms and number of practices that used any term at least once.

## Results

Three hundred sixty-nine websites were sampled according to the proportions provided by the Chiropractic Board of Australia. Due to minor errors in data collection, some states/territories were slightly oversampled, others slightly undersampled, however the overall national sample was achieved to reach the desired 95% confidence with 5% margin of error. See Table 1.

**Table 1:** Websites sampled, based on 5500 chiropractors in Australia

State/Territory	Percent of chiropractors according to CBA	Proportionate number to sample according to CBA percentage	Number actually sampled
ACT	1.3	5	5
NSW	33.5	124	96
NT	0.5	2	10
QLD	16.0	59	56
SA	7.0	26	33
TAS	1.0	4	24
VIC	25.9	96	93
WA	11.8	44	52
**TOTAL**	**97**^a^	**360**	**369**

Legend: *CBA* Chiropractic Board of Australia, the registration agency, *ACT* Australian Capital Territory, *NSW* New South Wales, *NT* Northern Territory, *QLD* Queensland, *SA* South Australia, *TAS* Tasmania, *VIC* Victoria, *WA* Western Australia

*3% of chiropractors did not disclose a principal place of practice to the CBA

All occurrences of each term were tallied for each state and territory. Adjust(−ing/−ment) was the most commonly occurring term at 2249 total occurrences nationwide, followed by wellness at 872, subluxation at 489, vital(−ism/−istic) at 158, and Innate at 137. See Table 2 for findings from each state and territory.

**Table 2:** All occurrences of each term by state/territory

State/ Territory	Subluxation	Vital(−ism/−istic)	Wellness	Innate	Adjust(−ing/−ment)	Totals
ACT	15	0	6	0	29	50
NSW	129	64	275	17	431	916
NT	44	9	8	1	62	124
QLD	83	23	255	9	712	1082
SA	97	21	85	14	330	547
TAS	9	14	35	49	96	203
VIC	83	20	158	46	337	644
WA	29	7	50	1	252	339
**TOTAL**	**489**	**158**	**872**	**137**	**2249**	**3905**

Legend: These numbers include multiple occurrences on each website. When a term was found on a website, it tended to be used multiple times

85% of Australian chiropractic websites use one or more chiropractic-specific terms. Nationwide, adjust(−ing/−ment) was most common with 283 (77%) websites using the term at least once, followed by wellness at 199 (33%), subluxation at 104 (28%), vital(−ism/−istic) at 71 (19%), and Innate at 39 (11%). See Table 3.

**Table 3:** Numbers of websites with any occurrence of the chiropractic-specific terms, n (%)

State/ Territory	Subluxation n (%)	Vital (−ism/−istic) n (%)	Wellness n (%)	Innate n (%)	Adjust (−ing/-ment) n (%)	Total websites with occurrence of any term n (%)	Number of websites sampled
ACT	3 (60)	0 (0)	2 (40)	0 (0)	5 (100)	5 (100)	5
NSW	26 (27)	30 (31)	55 (57)	16 (17)	71 (74)	82 (85)	96
NT	4 (40)	4 (40)	5 (50)	1 (10)	10 (10)	10 (100)	10
QLD	17 (30)	12 (21)	40 (50)	5 (9)	49 (88)	51 (91)	56
SA	12 (36)	10 (30)	20 (71)	8 (24)	29 (88)	31 (94)	33
TAS	6 (25)	3 (13)	11 (61)	2 (8)	17 (71)	20 (83)	24
VIC	25 (27)	10 (11)	49 (46)	6 (6)	62 (67)	74 (80)	93
WA	11 (21)	2 (4)	17 (53)	1 (2)	40 (77)	42 (81)	52
**TOTAL**	**104 (28)**	**71 (19)**	**199 (33)**	**39 (11)**	**283 (77)**	**315 (85)**	**369**

Legend: Whether a website mentioned a term one time or many, it was counted once for this table.

## Discussion

While any profession will have some amount of profession-specific terminology, chiropractic falls within the larger realm of health care professions, all of which should reasonably be expected to use a common terminology. 85% of Australian chiropractic websites sampled used one or more chiropractic-specific terms, representing chiropractic-specific concepts, would seem to indicate that a majority of Australian chiropractors view themselves as providing a service that is different to that of other health practitioners. Non-evidence-based beliefs have been found in Australian chiropractic students as well as in practitioners in other countries [57–60]. Since a variety of practitioners may provide manual therapy including manipulation, it is likely that this view is maintained by a combination of the persistence of traditional chiropractic beliefs like nerve interference, postural correction, etc., and deference to chiropractic history, taught in schools and seminars, and tolerated by regulatory bodies.

Since terms tended to occur multiple times when they appeared on any website, it was thought that the most useful metric would be the number of websites that mentioned any term any number of times. This represents the prevalence of chiropractic-specific terminology in the chiropractic community overall more accurately than the total number of times terms occurred.

77% of Australian chiropractors were found to use the term adjustment rather than the more generic manipulation. Unless there is something unusual about a chiropractic adjustment, some method or effect that is different from a manipulation delivered by a physiotherapist or osteopath, then there is no need for a different term. Some chiropractors argue that it is, in fact, different, that a chiropractic adjustment is specific and/or provides neurological/organic effects absent in the manipulations of other health care professionals [61–63]. However, there is little evidence of either specificity [64–68] or unique neurological/organic effects [69–72]. Therefore, there is little need for a chiropractic-specific term for manipulative therapy.

Strong emphasis on adjustment reduces chiropractic from a healthcare profession with a the broad range of diagnostic and therapeutic capabilities to a single therapeutic intervention. Chiropractors can provide various types of therapy, excluding drugs and surgery, but including expert advice on positive lifestyle changes [73]. Spinal manipulation is merely one tool in a large and diverse tool box that includes soft tissue therapy, rehabilitation techniques, patient education, exercise, and more. Chiropractors also receive considerable training in diagnosis [74]; the ability to differentiate an organic/pathological disorder that presents as musculoskeletal pain from a genuinely musculoskeletal disorder amenable to manual therapy should not be underestimated as a defining characteristic of the profession. It could be emphasised to administrators and policy-makers as a way to help them reduce the burden of musculoskeletal disorders on health care systems, whether governmental or private. The cost-effectiveness of using musculoskeletal experts in triage and treatment positions is now being recognised in the United Kingdom (UK), but physiotherapists are being awarded the First Contact Practitioner posts within the National Health Service [75].

The results for the terms subluxation, vital(−ism/−istic), and Innate mean that some Australian chiropractors publicly espouse terms that represent an alternative view of the mechanism of health and disease. This representation of anti-science separates chiropractic from mainstream health care, yet many within chiropractic would like to be viewed as mainstream and scientific [76, 77]. Codification of subluxation theory in American Medicare law has undoubtedly contributed to its perception by some as mainstream [13].

It would seem to require tolerance of cognitive dissonance to hold both scientific and vitalistic ideas at the same time. This has been found in at least one study [78]. Cognitive dissonance is a feeling of discomfort from maintaining two ideas simultaneously, both of which cannot be correct. For instance, it cannot be true that chiropractic adjustments to relieve vertebral subluxations are the key to maintaining health while so many people around the world who have never seen a chiropractor are living in perfect health. It seems unlikely that non-chiropractors, whether health professionals, representatives of the media, or lay public would tolerate such internal mental conflict so well.

The use of unique terms also allows detractors to generalise these concepts to all chiropractors [76, 79–81]. It has even been shown to prompt people to question the validity of complementary medicine altogether [79]. As noted previously, the presence of an alternative paradigm of health is divisive within chiropractic as well [24, 38, 82].

Wellness is probably the weakest term as regards chiropractic specificity. Chiropractors may use it in a profession-specific context, referring to the power of the adjustment in restoring health. In these circumstances, the term may be used in conjunction with the word chiropractic (e.g. “We promote chiropractic wellness,” or “We practice wellness chiropractic.”) [45] But wellness is a commonly-encountered term that is used in promoting public health initiatives such as stress reduction or moderation in alcohol intake, and thus wellness has a mainstream usage unrelated to chiropractic, as well. However, its use by chiropractors denoting broad effects to chiropractic treatment beyond pain relief and the improvement in quality-of-life that pain-reduced or pain-free daily activities provides, further indicates the self-separation of some chiropractors from mainstream health care.

Nelson, et al. noted the lack of cultural authority demonstrated by chiropractic because of the lack of scientific coherence in the unique chiropractic theories [83]. Similarly, lack of moral authority because of traditional beliefs persistent in homeopathy have threatened public funding and perceptions of legitimacy in that profession [84, 85]. Diversity in chiropractic care has been found to deter referrals from other professions to chiropractors and to be a source of internal conflict [10, 80]. Student populations at different educational institution have been found to use different lexicons, either more scientific or more traditional, depending on the institution, so tolerance for diversity in education seems to contribute to division as well [86].

Unique terminology and concepts also have a negative effect on research. If diagnostic methods, treatment effects, and outcome measures are not agreed among health care professions, little cross-professional investigation can be undertaken, as Johnson suggests would benefit patients as well as the profession of chiropractic [87]. Veziari et al. investigated barriers to research in complementary and alternative medicine (CAM) professions, separating them into two broad categories, capacity and culture [88]. Capacity included access, competency, bias, incentives and time. Culture included values and the complex system of CAM. Language/terminology would logically fall within culture but was not specifically investigated in the study.

Unique terminology and the non-evidence-based beliefs it represents are ongoing issues for the chiropractic profession, and solutions have been proposed. Nelson [83] as well as French, Downie, and Walker recommended abandonment of traditional chiropractic beliefs and adoption of a model for the profession centred on evidence-based spine care [89]. Triano et al. developed a plan in 2010, focusing on improving educational quality and integration with other health professions [90]. Johnson et al. noted that chiropractic was well-placed to take a greater role in public health issues [91]. Murphy et al. cited the mainstreaming of podiatry as an example and suggested that chiropractic adopt the key characteristics that helped that profession gain legitimacy and integration. They were: become involved in public health, reform education, acquire hospital-based residencies, establish a clear identity, and ensure fidelity to the social contract [92].

### Strengths

This study improves on previous studies of this type by searching for multiple chiropractic-specific terms, thus capturing a broader representation of uniquely chiropractic concepts. In addition, terms were examined to ensure that they were positively representing those concepts. 95% confidence interval was achieved with 5% margin of error.

### Limitations

Google results are not random and favour clinics that are more skilled with marketing; this may have affected the results, but in an unknown way. This was an Australian study, so the results cannot be generalised throughout the world, as other chiropractic communities may hold different values. There are various ways of expressing these ideas search and limiting the search to five terms means that it is likely that not all clinics expressing chiropractic-specific concepts were captured. There have been increased efforts at compliance with advertising standards in Australia in the past few years [93] and this may have led some chiropractors not to abandon their beliefs but rather to mask their expression of them to adhere to CBA standards; therefore, chiropractic-specific ideas held by practitioners may be underrepresented in this study. Not every registered chiropractor has a website; some may work in a group practice with a group website; some may only use social media; some may not be represented on the internet at all, so some chiropractors may have been excluded from sampling. Thus, the results may not be generalisable to the chiropractor population of Australia. Only one research assistant made judgments on the websites in their assigned state or territory, so some terms may have been misinterpreted.

## Conclusion

A majority of the Australian chiropractors sampled used one or more chiropractic-specific terms on their websites. Future research should explore the effects of chiropractic language on the public, policy-makers, and other health care professionals. Given the apparent detrimental effects, chiropractic should consider abandoning unique language.

## Data Availability

Not applicable.
